# Hsa_circ_0134111 promotes intervertebral disc degeneration via sponging miR-578

**DOI:** 10.1038/s41420-022-00856-2

**Published:** 2022-02-08

**Authors:** Peng Yan, Chong Sun, Liangrui Luan, Jialuo Han, Yang Qu, Chuanli Zhou, Derong Xu

**Affiliations:** Department of Orthopedic Surgery, The Affiliated Hospital of Qingdao University, 266000 Qingdao, Shandong China

**Keywords:** miRNAs, Cell growth

## Abstract

Intervertebral disc degeneration (IDD) is a chronic degenerative and age-dependent process characterized by aberrant apoptosis, proliferation, synthesis, and catabolism of the extracellular matrix of the nucleus pulposus (NP) cells. Recently, studies showed that circular RNAs play important roles in the development of many diseases. However, the role of circRNAs in IDD development remains unknown. We showed that circ_0134111 level was overexpressed in IDD tissue samples as compar-ed to control tissues. The upregulation of circ_0134111 was more drastic in the moderate and severe IDD cases than in those with mild IDD. In addition, we showed that interleukin-1β and tumor necrosis factor-α exposure significantly enhanced circ_0134111 expression in NP cells. Furthermore, ectopic expression of circ_0134111 induced proliferation, pro-inflammatory cytokine secretion, and ECM degradation in the NP cells. We also showed that circ_0134111 directly interacted with microRNA (miR)-578 in NP cells where elevated expression of circ_0134111 enhanced the ADAMTS-5 and MMP-9 expression. Moreover, miR-578 expression was significantly decreased in IDD patients and the miR-578 expression was negatively correlated with circ_0134111 expression in the IDD samples. Interleukin-1β and tumor necrosis factor-α exposure significantly decreased miR-578 levels in NP cells, in which ectopic miR-578 expression inhibited cell growth, pro-inflammatory cytokine expression, and ECM degradation. Finally, we showed that circ_0134111 overexpression induced the IDD-related phenotypic changes through inhibiting miR-578. These data suggested that circ_0134111 could promote the progression of IDD through enhancing aberrant NP cell growth, inflammation, and ECM degradation partly via regulating miR-578.

## Introduction

Low back pain (LBP) is a leading cause of physical disability and is one of the most frequently encountered health problems in clinics, causing substantial global public health and economic burden [1–4]. IDD is the commonest cause of LBP [5, 6]. Intervertebral discs are composed of three interrelated structures: annulus fibrosus; cartilaginous endplates and nucleus pulposus (NP). IDD is associated with injury of the adjacent structures, which leads to functional impairment and clinical symptoms including myelopathy, back pain, and radiculopathy [7–10]. IDD is a chronic degenerative and age-dependent process where aberrant NP cell apoptosis, proliferation, and extracellular matrix (ECM) catabolism/anabolism occur [11–15]. However, the detailed molecular mechanisms contributing to these phenotypic changes remain unclear. Thus, it is imperative to delineate these mechanisms so as to identify potential therapeutic targets in IDD.

Recently, noncoding RNAs, including microRNAs, long noncoding RNAs, and circRNAs (circular RNAs) have been shown to act important roles in the development of many diseases [16–20]. CircRNAs are one relatively novel noncoding RNAs type that is abundantly expressed in mammals [21–24]. Mechanistically, circRNAs modulate gene expression via transcriptional or post-transcriptional mechanisms, including sponging of miRNAs to regulate the downstream signaling axes [23, 25–27]. Growing evidence suggested that circRNAs play critical roles in cell functions, such as apoptosis, growth, metabolism, and ECM synthesis [28, 29] circRNAs have also been promulgated as therapeutic targets in different diseases including neurological dysfunction, metabolic diseases, cancers, and cardiovascular diseases [30–33]. For instance, a recent study showed that circ_0134111 could induce osteoarthritis development through regulating miR-224-5p/CCL1 and miR-515-5p-SOCS1 axes [34].

Our study identified circ_0134111 as one of the most highly upregulated circRNAs in the IDD tissue samples as compared to control specimens. Upstream, pro-inflammatory cytokines TNF-α and IL-1β were found to significantly increase circ_0134111 expression. Furthermore, enforced expression of circ_0134111 induced aberrant ECM degradation, proliferation, and inflammatory cytokine secretion in NP cells.

## Results

### circ_0134111 expression was increased in IDD tissues

**Fig. 1:**
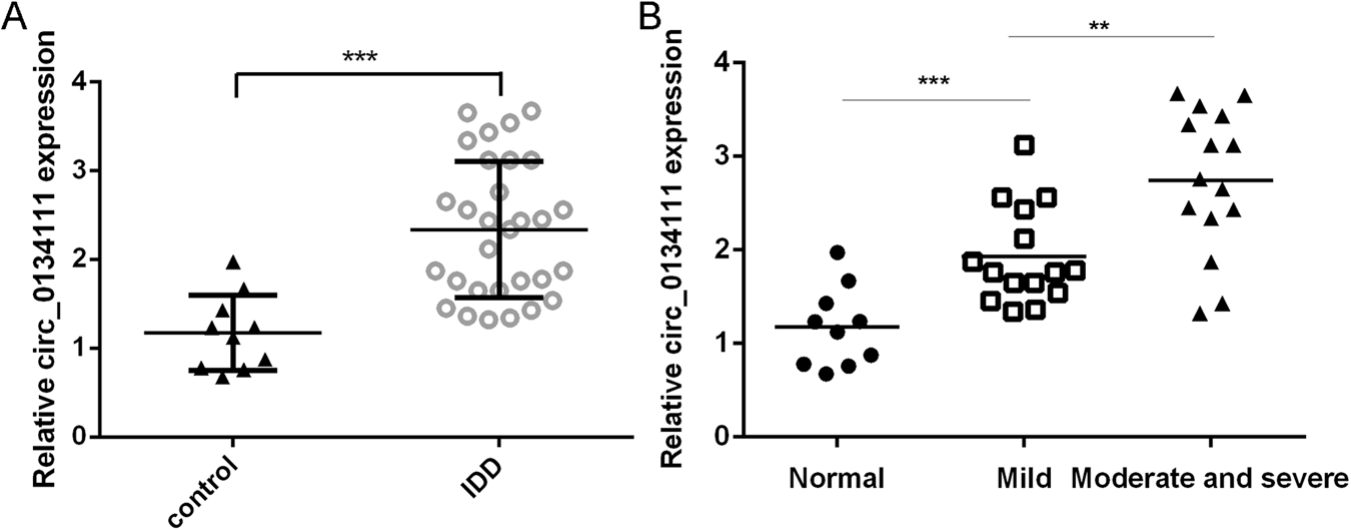
circ_0134111 expression was increased in IDD tissues. **A** The expression of circ_0134111 in disc tissues collected from 30 IDD patients and 10 control subjects was measured by qRT-PCR. **B** The expression of circ_0134111 was higher in the moderate/severe than the normal IVD tissues or the mild group. **p < 0.01 and ***p < 0.001.

To explore whether circ_0134111 is deregulated in IDD, its expression level was measured by qRT-PCR in 30 IDD tissues and 10 control disc samples. The expression of circ_0134111 was higher in the IDD samples than in the control tissues (Fig. 1A). Furthermore, the extent of circ_0134111 upregulation was highest in the moderate/severe group than the mild group (Fig. 1B).

### IL-1β and TNF-α induced circ_0134111 expression in NP cells

**Fig. 2:**
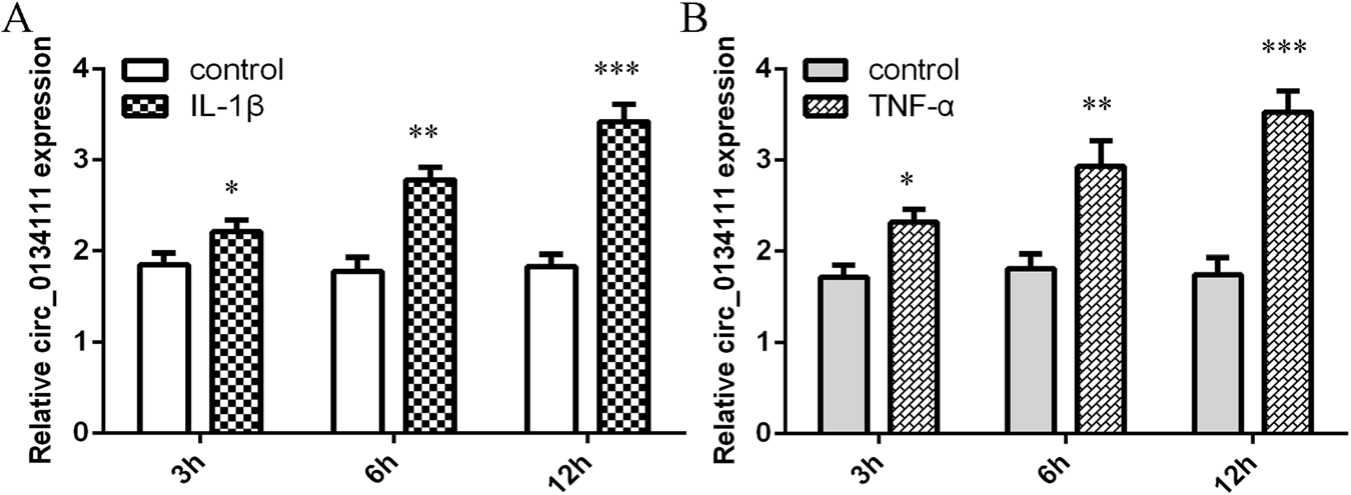
IL-1β and TNF-α induced circ_0134111 expression in NP cells. **A** IL-1β significantly increased circ_0134111 expression in NP cells. **B** The expression of circ_0134111 in NP cells after incubation with TNF-α. *p < 0.05, **p < 0.01, and ***p < 0.001.

circ_0134111 expression level in NP cells after exposure to two important pro-inflammatory cytokines, namely IL-1β and TNF-α, was measured by qRT-PCR. As shown in Fig. 2A, IL-1β significantly increased circ_0134111 expression in NP cells. The expression of circ_0134111 was also upregulated in NP cells after exposure to TNF-α (Fig. 2B).

### circ_0134111 directly interacted with miR-578 in NP cells

**Fig. 3:**
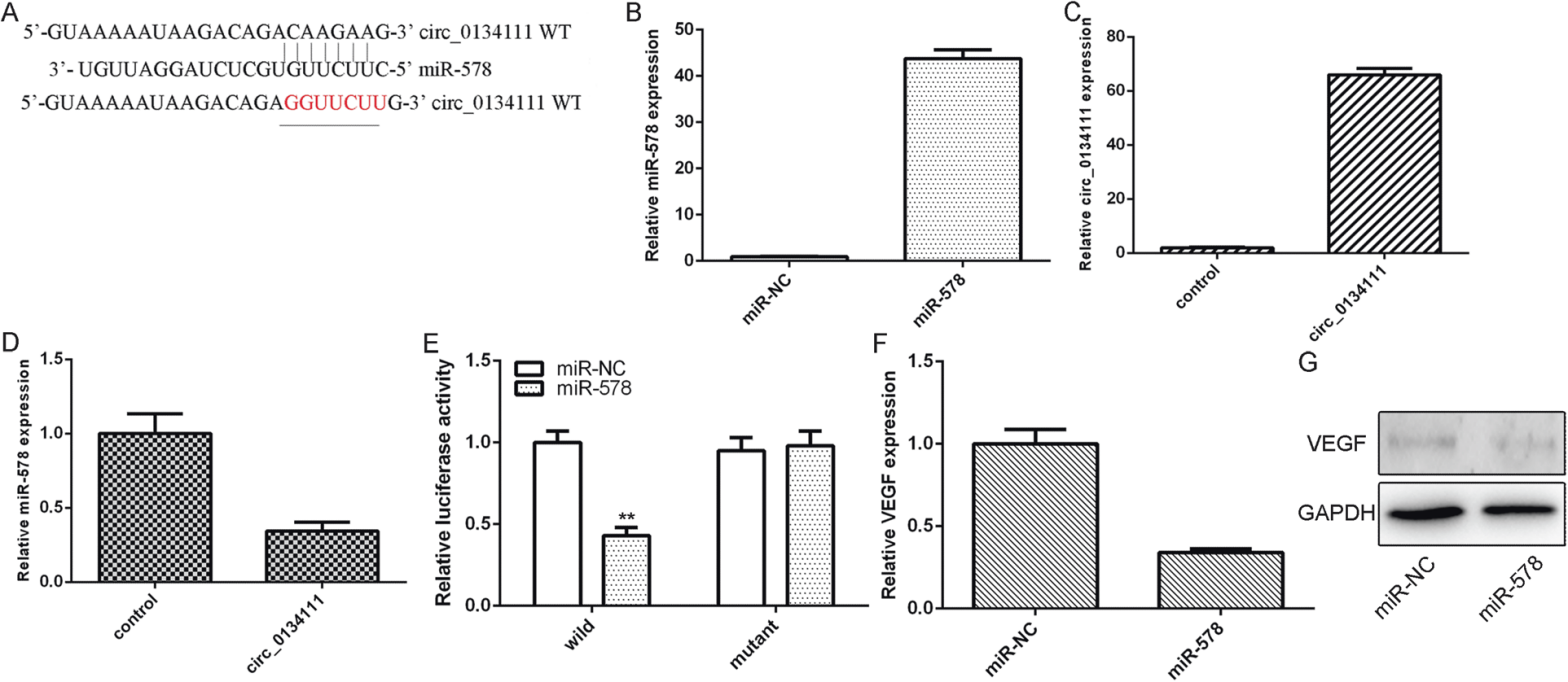
circ_0134111 directly interacted with miR-578 in NP cells. **A** miR-578 harbored a circ_0134111-binding site as predicted using bioinformatics. **B** The expression of miR-578 was upregulated in NP cells after transfection with miR-578 mimic. **C** The circ_0134111 expression was increased in NP cells after transfection with pcDNA-circ_0134111 vector. **D** Enforced expression of circ_0134111 reduced miR-578 expression. **E** The luciferase activity of wild-type but not the bind site-mutated circ_0134111 was downregulated upon transfection with miR-578 mimic. **F** Overexpression of miR-578 suppressed VEGF expression in the NP cells. **G** The protein expression of VEGF was measured by western blots. **p < 0.01.

To investigate the downstream signaling of circ_0134111, bioinformatics tools were used to predict the downstream targets. As displayed in Fig. 3A, miR-578 harbored a circ_0134111-a binding site. We, therefore, designed experiments to validate their potential functional interaction. The expression of miR-578 was upregulated in NP cells after transfection of the miR-578 mimic (Fig. 3B). circ_0134111 expression was higher in NP cells after transfection of the pcDNA-circ_0134111 vector (Fig. 3C). Overexpression of circ_0134111 reduced miR-578 expression (Fig. 3D) whereas luciferase reporter assay showed that the luciferase activity of wild-type but not the binding site-mutated circ_0134111 was downregulated upon transfection of miR-578 mimic, indicating the interaction between miR-578 and circ_0134111 (Fig. 3E). Furthermore, miR-578 overexpression suppressed the VEGF expression (a known target of miR-578) in NP cells (Fig. 3F, G).

### miR-578 level was significantly downregulated in IDD tissues

**Fig. 4:**
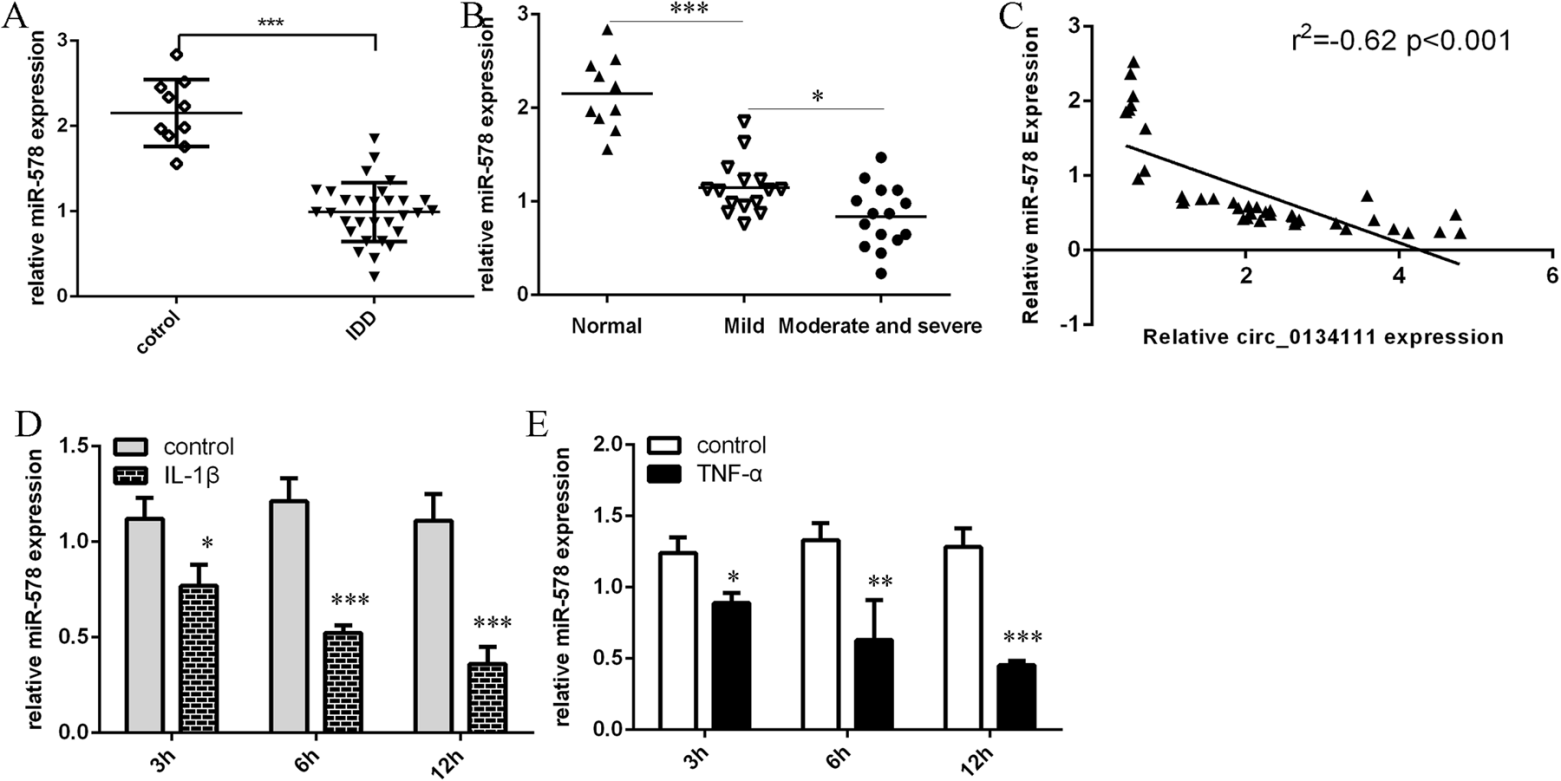
miR-578 expression was significantly decreased in IDD tissues. **A** miR-578 expression was lower in the IDD tissues compared to control tissues. **B** miR-578 expression was downregulated in the moderate/severe group compared to normal tissues or the mild group. **C** The expression of miR-578 was negatively correlated with circ_0134111 expression in the IDD samples. **D** IL-1β significantly decreased the miR-578 expression in NP cells. **E** The expression of miR-578 was downregulated in the NP cells after treatment with TNF-α. *p < 0.05, **p < 0.01, and ***p < 0.001.

To study whether miR-578 expression was changed in IDD, miR-578 expression was determined with qRT-PCR in disc tissues collected from the same cohort of IDD patients and 1control subjects. As shown in Fig. 4A, the miR-578 expression level was lower in the IDD tissues as compared to the control samples. Moreover, the miR-578 expression level was lowest in the moderate/severe group (Fig. 4B). Furthermore, miR-578 was negatively correlated with circ_0134111 level in the IDD samples (Fig. 4C). The miR-578 expression level in NP cells after IL-1β and TNF-α exposure was significantly decreased (Fig. 4D, E).

### circ_0134111 overexpression induced proliferation, pro-inflammatory cytokine secretion, and ECM degradation in NP cells

**Fig. 5:**
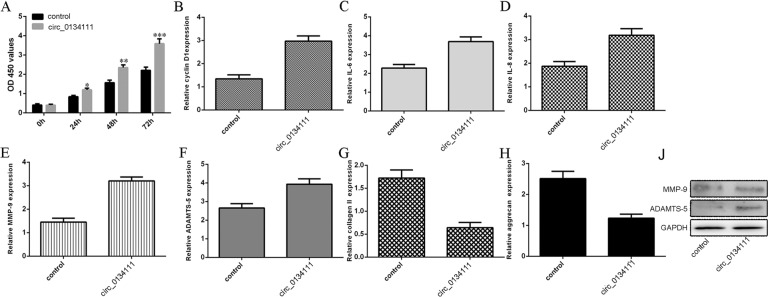
circ_0134111 overexpression induced proliferation, pro-inflammatory cytokine secretion, and ECM degradation in NP cells. **A** circ_0134111 overexpression promoted NP cell proliferation as shown by the CCK-8 assay. **B** Ectopic expression of circ_0134111 increased the cyclin D1 expression. **C–F** Overexpression of circ_0134111 induced **C** IL-6, **D** IL-8, **E** MMP-9, and **F** ADAMTS-5 expression as shown by qRT-PCR assay. **G, H** The expression of type II collagen and aggrecan was determined by qRT-PCR assay. Ectopic expression of circ_0134111 decreased **G** type II collagen and **H** aggrecan expression in NP cells. **J** The protein expression of MMP-9 and ADAMTS-5 was measured by western blots. *p < 0.05, **p < 0.01, and ***p < 0.001.

circ_0134111 overexpression promoted NP cell proliferation as shown by the CCK-8 assay (Fig. 5A). In line with this, ectopic expression of circ_0134111 increased cyclin D1 expression in NP cells (Fig. 5B). In addition, overexpression of circ_0134111 induced IL-6 and IL-8 expression as shown by qRT-PCR (Fig. 5C, D). Moreover, enforced expression of circ_0134111 enhanced the mRNA level of MMP-9 and ADAMTS-5, both of which are IDD-related ECM-degrading enzymes, in NP cells (Fig. 5E, F). We also demonstrated that ectopic circ_0134111 expression suppressed the aggrecan and type II collagen expression (Fig. 5G, H). Consistent with the change of gene expression at the mRNA level, overexpression of circ_0134111 promoted MMP-9 and ADAMTS-5 protein expression in NP cells (Fig. 5J).

### Ectopic miR-578 expression inhibited proliferation, pro-inflammatory cytokine secretion, and ECM degradation

**Fig. 6:**
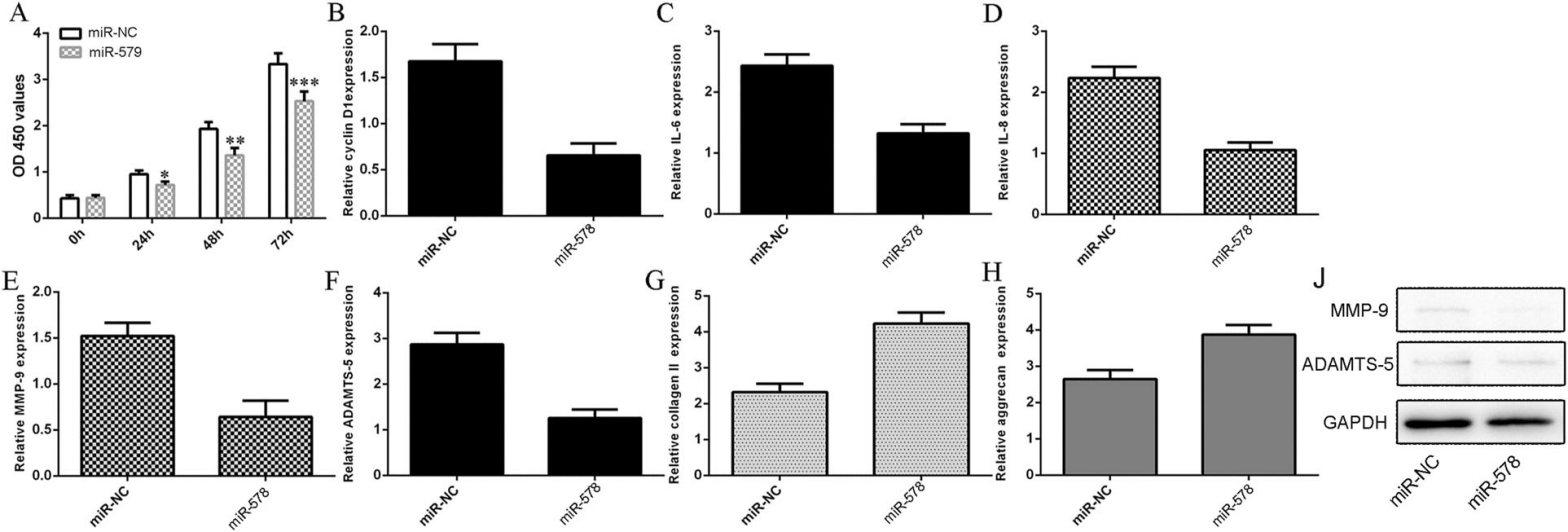
Ectopic expression of miR-578 inhibited proliferation, pro-inflammatory cytokine secretion, and ECM degradation in NP cells. **A** miR-578 overexpression inhibited NP cell proliferation as shown by the CCK-8 assay. **B** The expression of cyclin D1 was analyzed by using qRT-PCR and GAPDH was used as the internal control. **C–F** Overexpression of miR-578 suppressed **C** IL-6, **D** IL-8, **E** MMP-9, and **F** ADAMTS-5 expression as shown by qRT-PCR. **G, H** The expression of type II collagen and aggrecan was determined by qRT-PCR assay. Ectopic expression of circ_0134111 decreased **G** type II collagen and **H** aggrecan expression in NP cells. **J** The protein expression of MMP-9 and ADAMTS-5 was measured by western blots. *p < 0.05, **p < 0.01, and ***p < 0.001.

Opposite to the actions of circ_0134111, miR-578 overexpression inhibited NP cell proliferation (Fig. 6A), decreased cyclin D1 expression (Fig. 6B), suppressed IL-6 and IL-8 RNA expression (Fig. 6C, D), and inhibited the mRNA expression of MMP-9 and ADAMTS-5 in NP cells (Fig. 6E, F). Consistently, ectopic expression of miR-578 enhanced the type II collagen and aggrecan expression (Fig. 6G, H) and inhibited MMP-9 and ADAMTS-5 protein expression in NP cells (Fig. 6J).

### circ_0134111 produced IDD-related phenotypes through sponging miR-578

**Fig. 7:**
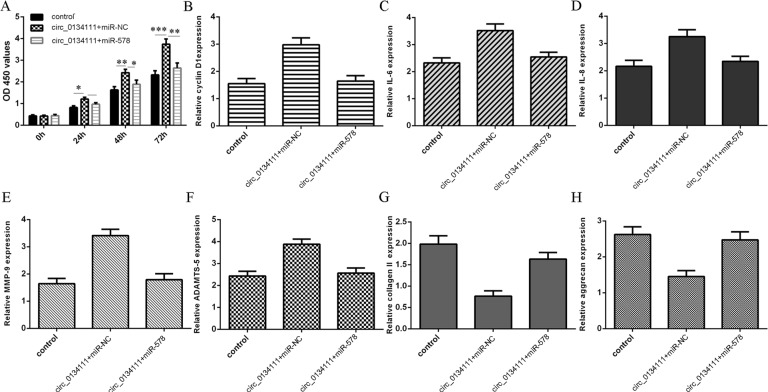
circ_0134111 regulated proliferation, pro-inflammatory cytokine secretion, and ECM degradation of NP cells through miR-578. **A** Cell growth was meaured by CCK-8 assay. **B** The level of cyclin D1 was measured by qRT-PCR. **C, D** miR-578 overexpression decreased IL-6 and IL-8 level in the circ_0134111-overexpressing NP cells. The level of IL-6 and IL-8 was detected using qRT-PCR. **E, F** Ectopic miR-578 expression decreased MMP-13 and ADAMTS-5 expression in the circ_0134111-overexpressing NP cells. MMP-13 and ADAMTS-5 expression was detected using qRT-PCR. **G, H** miR-578 overexpression promoted the expression of type II collagen and aggrecan in circ_0134111-overexpressing NP cells. Type II collagen and aggrecan level was detected by qRT-PCR. *p < 0.05, **p < 0.01, and ***p < 0.001.

We further studied whether circ_0134111 regulated proliferation, cytokine secretion, and ECM degradation through regulating miR-578 expression in NP cells. We found that overexpression of circ_0134111 promoted cell proliferation in NP cells, where miR-578 reversed this effect (Fig. 7A). Overexpression of miR-578 also suppressed the cyclin D1 expression induced by circ_0134111 overexpression (Fig. 7B). Similarly, miR-578 decreased circ_0134111-induced upregulation of IL-6 (Fig. 7C), IL-8 (Fig. 7D), MMP-9 (Fig. 7E), and ADAMTS-5 (Fig. 7F) in NP cells. miR-578 overexpression reversed the downregulation of type II collagen and aggrecan in the circ_0134111-overexpressing NP cells (Fig. 7G, H).

## Discussion

We found that the circ_0134111 level was upregulated in IDD tissues and the upregulation of circ_0134111 correlated with the clinical severity. Upstream, we showed that two pro-inflammatory cytokines—IL-1β and TNF-α could induce circ_0134111 expression in NP cells. Functionally, circ_0134111 induced NP cell proliferation, pro-inflammatory cytokine secretion, and ECM degradation whereas miR-578 produced the opposite effects. Mechanistically, circ_0134111 directly interacted with miR-578 to mediate the phenotypic changes. These data suggested that circ_0134111 could promote IDD progression, at least in part, through regulating miR-578 expression to alter NP cell functions.

Recently, several circRNAs play crucial roles in IDD development. For example, Hu et al. [35] showed that circ_0022382 ameliorated IDD through modulating TGF-β3 expression by sponging miR-4726-5p. Zhang et al. [36] also demonstrated that circSNHG5 inhibited cartilage endplate degradation through sponging mir-495-3p to derepress CITED2. Wang et al. [37] reported that circARL15 modulated IDD progression via regulating the miR-431-5p/DISC1 axis. Zhang et al. [38] demonstrated that circ_ITCH induced ECM degradation through regulating Wnt/β-catenin signaling in IDD. Huang et al. [39] found that circPKNOX1 suppressed IDD development through regulating the miR-370-3p/KIAA0355 axis. Chen et al. [40] also found that circ-GLCE alleviated IDD development through modulating apoptosis and ECM degradation via targeting the miR-587/STAP1 axis. Recently, a new circRNA circ_0134111 has been identified to be involved in the development of osteoarthritis. Wu et al. [41] demonstrated that knockdown of circ_0134111 relieved IL-1β-induced inflammation, apoptosis, and ECM degradation through the miR-515-5p/SOCS1 axis in the human chondrocytes. Zhang et al. [42] showed that knockdown of circ_0134111 alleviated osteoarthritis symptoms through sponging miR-224-5p. Liu et al. [34] also found that circ_0134111 induced osteoarthritis development through modulating the miR-224-5p/CCL1 axis. In our study, we found that the same circRNA was upregulated in IDD tissue and its overexpression promoted aberrant NP cell phenotypes, including proliferation, cytokine expression, and ECM degradation

Numerous studies have suggested that circRNAs mediate their biological functions in IDD via sponging miRNAs. For example, exosome-transported circ_0000253 promoted IDD development via regulating miR-141-5p [43]. Guo et al. [44] demonstrated that FAM169A modulated IDD development through sponging miR-583. Cui et al. [45] showed that circ_001653 modulated ECM synthesis and cell proliferation through sponging miR-486-3p in IDD. Xie et al. [46] found that circERCC2 ameliorated IDD through modulating apoptosis and mitophagy via sponging miR-182-5p. We showed that circ_0134111 directly interacted with miR-578 in NP cells. Importantly, miR-578 expression was significantly downregulated in IDD tissues and was negatively correlated with circ_0134111 in the IDD samples. Finally, we confirmed that circ_0134111 overexpression induced the IDD-related phenotypes of NP cells through inhibiting miR-578. A previous study showed that miR-578 could target VEGF expression to suppress osteosarcoma cell migration and proliferation [47]. In line with this, we found that overexpression of miR-578 inhibited VEGF expression, suggesting that circ_0134111 may regulate NP cell function via targeting miR-578/VEGF axis. Nevertheless, more experiments are needed to confirm the functional involvement of VEGF in the pathogenic action of circ_0134111.

In summary, our results demonstrated that circ_0134111 is aberrantly upregulated in the IDD tissues. The expression of this circRNA could also be induced by IL-1β and TNF-α in NP cells. circ_0134111 also alters NP cell phenotypes that are known to contribute to IDD progression. Mechanistically, miR-578 is the downstream target of circ_0134111. Our data suggested that circ_0134111 may be a novel therapeutic target in IDD.

## Materials and methods

### Sample collection

The intervertebral disc samples from the IDD patients and normal intervertebral disc samples from those with spondylolysis were collected from our hospital. These specimens were snap-frozen and stored in the liquid nitrogen until protein or RNA extraction.

### RNA extraction and quantitative RT-PCR

Total cellular and tissue RNA was extracted using Trizol (Life, CA, USA). Expression of lncRNA and mRNA was detected by qRT-PCR using the SYBR Green PCR mix on the BioRad IQ5 PCR system. GAPDH and U6 nuclear RNA were utilized as controls for mRNA/lncRNA and miRNA, respectively. These primer sequences are as follows: circ_0134111, forward 5’- GAAAACAGATGAGGAGAAGGCC-3’ and reverse 5’- CGTCTTTTTCTCAGCTTTGCC-3’; IL-6, forward 5’-GACTGATGTTGCTGACAGCCACTGC-3’ and reverse 5’-TAGCCACTGCTTCTGTGACTCTAACT-3’; IL-8, forward 5’-AAACCACCGGAAGGAACCAT-3’ and reverse 5’-GCCAGCTTGGAAGTCATGT-3’. VEGF, forward: 5′-GGACCCGAT GCGGTTAGAG-3′and reverse 5′-ATCAAGTGGATGCCCCACAG-3′;

### Cell culture and transfection

NP cells were separated and cultured according to previous studies. In brief, NP tissues were dissected for digestion with collagenase II in Dulbecco’s modified Eagle’s Medium (Life Technologies). NP cells were cultured in DMEM supplement with fetal bovine serum (FBS), streptomycin, and penicillin. circ_0134111 and control plasmid, miR-578 mimic and miR-NC were purchased from Genechem (Shanghai, China) and were transfected into cells using Lipofectamine2000 (Invitrogen, USA) according to the instructions of the manufacturer.

### Cell proliferation

After transfecting, cells were seeded in 96-well plates with the density at 5 × 10^3^ cells/well and cultured for different times (0, 24, 48, and 72 h). Ten microliters CCK-8 (Cell Counting Kit-8, DOJINDO) solution was added in each well and continued to incubate for 2 h at 37 °C. The absorbance at 450 nM was read on the microtiter reader.

### Western blots

Isolation of total protein from IDD tissues or NP cells was performed with RIPA buffer. The concentration of protein was determined with the bicinchoninic acid (BCA) protocol. An equal amount of protein was resolved by SDS-polyacrylamide gel electrophoresis (Invitrogen, USA) and transferred to the PVDF membrane (Millipore). After blocking with 5% milk, the membrane was incubated with primary antibody (anti-MMP-9, No. MA5-15886; anti-ADAMTS-5, No. PA5-14350; Invitrogen). After washing three times with TBST, the membrane was incubated with an HRP-conjugated secondary antibody. The signals were generated with the chemiluminescent reagents. The primary antibodies used in this study are as follows: VEGF, MMP-9, ADAMTS-5, and GAPDH (Santa Cruz Biotechnology).

### Statistical analysis

Results were shown as the means ± standard deviation (SD). All statistical tests were conducted using the SPSS 18.0 software (Chicago, USA). The significance of the difference between groups was determined using Student’s *t*-test or one-way ANOVA where appropriate. *P* < 0.05 was considered as significant.

## Data Availability

Research data are not shared.
